# Supplementary material for: Evolution of antiviral resistance captures a transient interdomain functional interaction between chikungunya virus envelope glycoproteins
Source: bioRxiv. 2024 Nov 11:2024.11.11.623010. Preprint. [Version 1] doi: 10.1101/2024.11.11.623010 (PMC11601244; doi:10.1101/2024.11.11.623010)
Supplement: Supplement 1 [file NIHPP2024.11.11.623010v1-supplement-1.pdf]

bioRxiv preprint doi: https://doi.org/10.1101/2024.11.11.623010; this version posted November 11, 2024. The copyright holder for this preprint (which was not certified by peer review) is the author/funder, who has granted bioRxiv a license to display the preprint in perpetuity. It is made available under aCC-BY 4.0 International license.

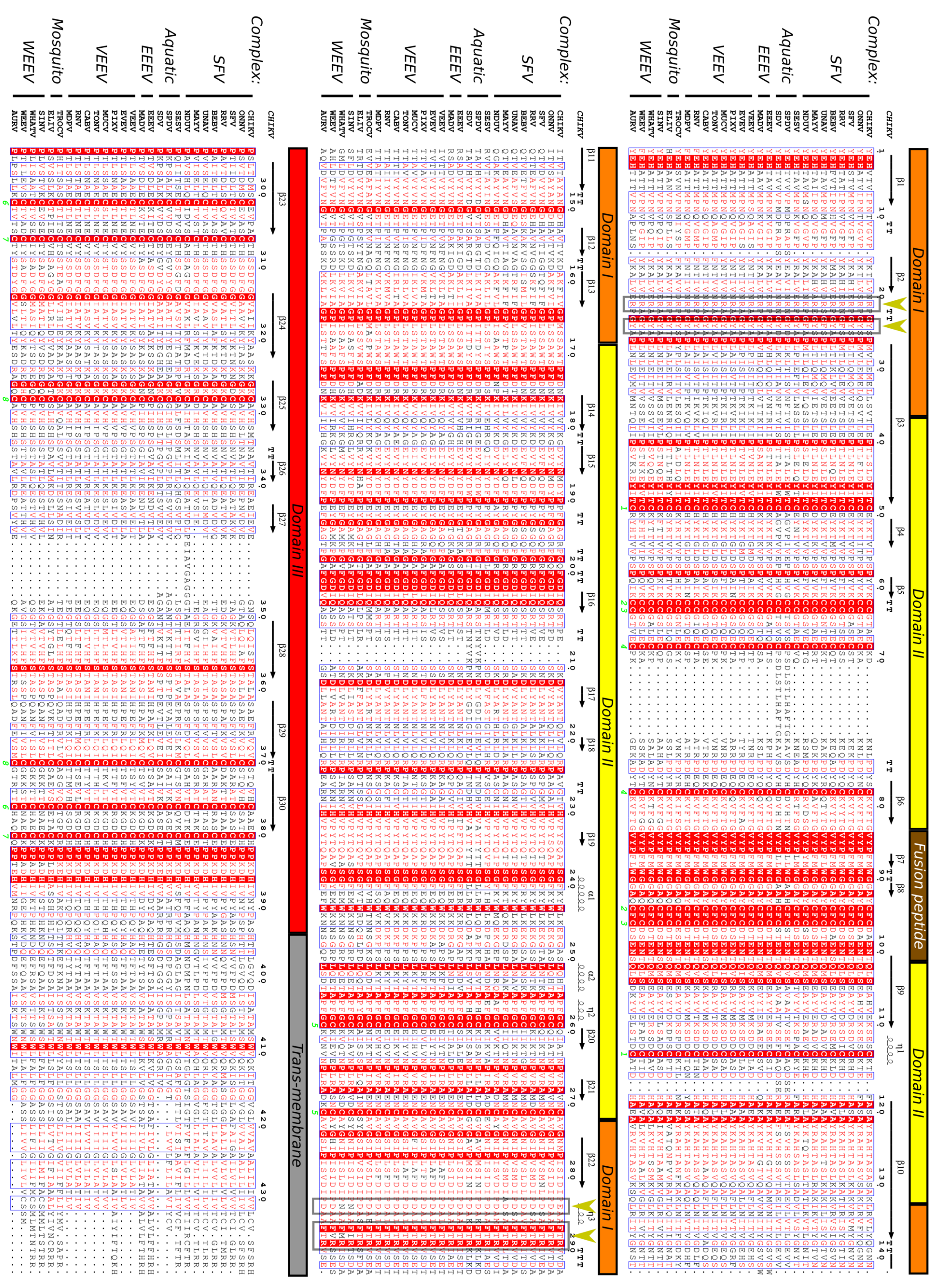

bioRxiv preprint doi: https://doi.org/10.1101/2024.11.11.623010; this version posted November 11, 2024. The copyright holder for this preprint (which was not certified by peer review) is the author/funder, who has granted bioRxiv a license to display the preprint in perpetuity. It is made available under aCC-BY 4.0 International license.

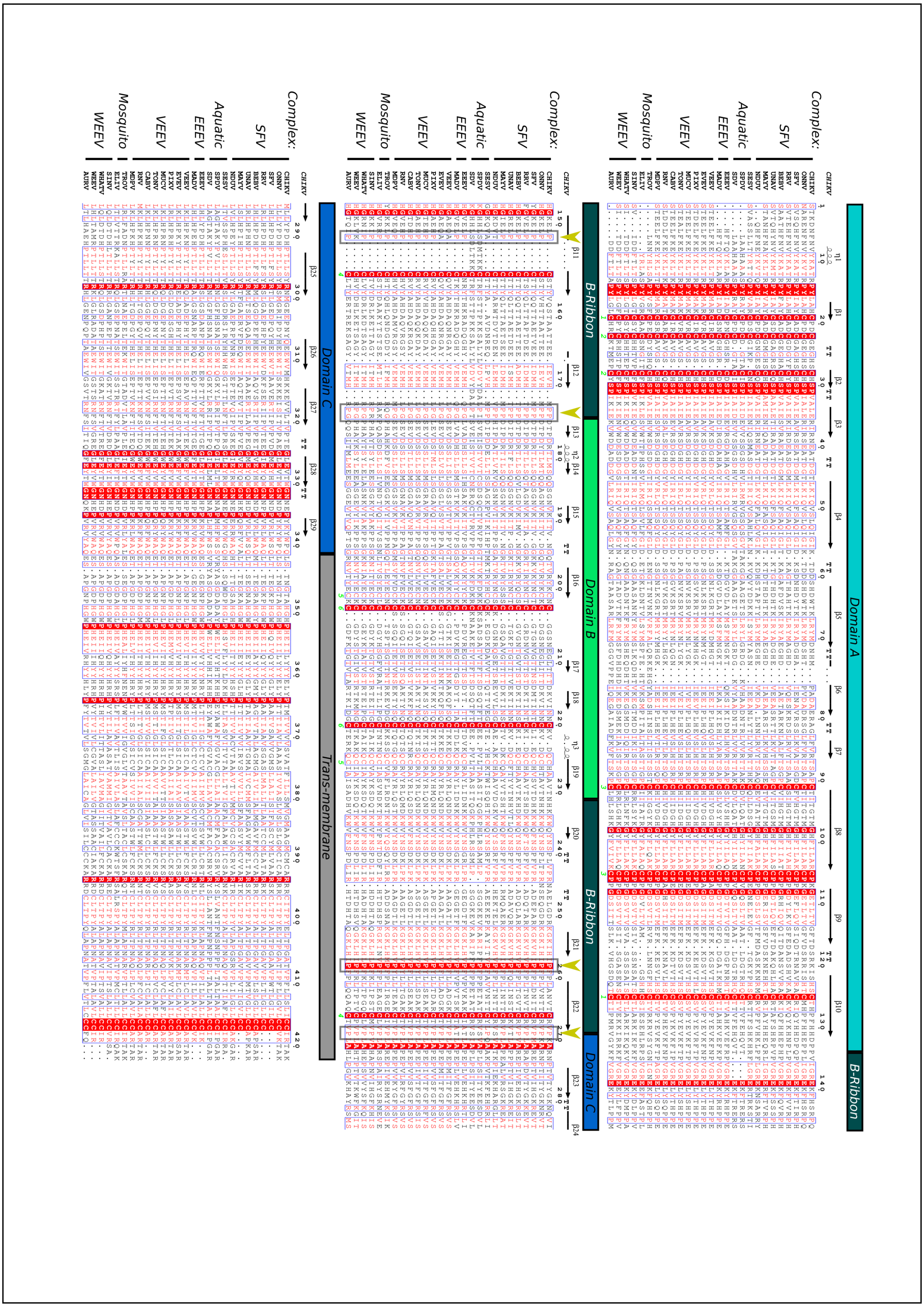

bioRxiv preprint doi: https://doi.org/10.1101/2024.11.11.623010; this version posted November 11, 2024. The copyright holder for this preprint (which was not certified by peer review) is the author/funder, who has granted bioRxiv a license to display the preprint in perpetuity. It is made available under aCC-BY 4.0 International license.

Supplementary Figure 1. Amino acid alignment of alphavirus E1 and E2 envelope proteins. Alignments were generated using MAFFT (44) and visualized with ESPript (45). Strictly conserved residues are highlighted with a red background. Disulfide bonds are indicated with a green number below the corresponding cysteine residue. Conserved residues are displayed in red characters and boxed in blue. Protein domains depicted above the alignment are colored as defined in Figure 1 and symbols correspond to the secondary structure of the protein as determined in (9): arrows represent β-strands and coils represent either alpha helices (α) or 3/10 helices (η). Arrowheads in the alignment of E2 point to prolines associated with arches in the complementary strands of the β-ribbon. In the alignment of E1, arrowheads point to interacting residues between E1-Y24 loop and the flexible linker between domains E1-I and E1-III. Alphavirus complexes are indicated on the left as follows, Semliki Forest complex (SFV): Chikungunya virus IOL (CHIK IOL, JF274082.1), O'nyong-nyong virus (ONNV, NC_001512.1), Semliki forest virus (SFV, NC_003215.1), Ross River virus (RRV, GQ433354.1), Bebaru virus (BEBV, HM147985.1), Una virus (UNAV, HM147992.1), Mayaro virus (MAYV, NC_003417.1), Ndumu virus (NDUV, JX644166.1); aquatic virus complex (Aquatic): Southern elephant seal virus (SESV, NC_016960.1), Salmon pancreas disease virus (SPDV, NC_003930.1), Sleeping disease virus (SDV, NC_003433.1); Eastern Equine Encephalitis complex (EEEV): Eastern equine encephalitis virus (EEEV, NC_003899.1), Madariaga virus (MADV, KJ469622.1); Venezuelan Equine Encephalitis complex (VEEV): Venezuelan equine encephalitis virus (VEEV, NC_001449.1), Everglades virus (EVEV, NC_038671.1), Pixuna virus (PIXV, NC_038673.1), Mucambo virus (MUCV, AF075253), Tonate virus (TONV, NC_038675.1), Cabassou virus (CABV, NC_038670.1), Rio Negro virus (RNV, NC_038674.1), Mosso das Pedras virus (MDPV, NC_038857.1); mosquito-specific virus complex (Mosquito): Trocara virus (TROV, NC_043402.1), Eilat virus (EILV, NC_018615.1); Western Equine Encephalitis complex (WEEV): Sindbis virus (SINV, NC_001547.1), Whataroa virus (WHATV, NC_016961.1), Western equine encephalomyelitis virus (WEEV, NC_003908.1), Aura virus (AURV, NC_003900.1).
